# Transition Pathways Out of Pediatric Care and Associated HIV Outcomes for Adolescents Living With HIV in South Africa

**DOI:** 10.1097/QAI.0000000000002125

**Published:** 2019-06-20

**Authors:** Roxanna Haghighat, Elona Toska, Lucie Cluver, Laurie Gulaid, Daniella Mark, Anurita Bains

**Affiliations:** a Department of Social Policy and Intervention, University of Oxford, Oxford, United Kingdom;; b AIDS and Society Research Unit, University of Cape Town, Cape Town, South Africa;; Departments of c Sociology;; d Psychiatry and Mental Health, University of Cape Town, Cape Town, South Africa;; e UNICEF Eastern and Southern Africa Regional Office, Johannesburg, South Africa;; f Pediatric-Adolescent Treatment Africa, Cape Town, South Africa;; g Department of Psychology, University of Cape Town, Cape Town, South Africa; and; h UNICEF Eastern and Southern Africa Regional Office, Nairobi, Kenya.

**Keywords:** adolescent, transition, HIV, youth, antiretroviral, sub-Saharan Africa

## Abstract

Research on adolescent transitions out of pediatric HIV care has focused on high-income countries, with limited understanding of transitions in sub-Saharan Africa's public health sector.

**Methods:**

Patient file data were extracted through December 2017 for all 10- to 19-year olds ever initiated on antiretroviral therapy in a health district of the Eastern Cape, South Africa (n = 951). Pathways in HIV care were identified by tracing movements across facility care types and levels. Associations between pathways and viral failure, mortality, loss to follow-up, and viral load change were tested in sequential multivariable regressions. Analyses controlled for sociodemographic and treatment-related variables. Thematic analyses of semistructured health care provider interviews identified transition support at included facilities.

**Results:**

Only 57.8% of adolescents had initiated antiretroviral therapy in pediatric care, and 20.4% of the total cohort had transitioned out of pediatric HIV care. Among the 42.2% who had initiated in nonpediatric care, 93.8% remained exclusively in nonpediatric care. Median age at first transition was 14 years. Two main pathways were identified: *classical transition* to adult HIV care (43.3%) and *down referral transition* to primary health care clinics (56.7%). Across pathways, 27.3% experienced *cyclical transition* or repeated movement between pediatric and nonpediatric care. Independent of covariates, adolescents with *down referral transition* were less likely to demonstrate viral failure (adjusted odds ratio, 0.21; 95% confidence interval: 0.10 to 0.42; *P* < 0.001). Mortality and loss to follow-up were not associated with either pathway. Median posttransition viral load change was not clinically significant (median, 0.00; interquartile range: 0.00–0.35) or associated with transition pathways. Health care providers described informal “protocols” for mitigating risk of negative posttransition HIV outcomes.

**Conclusions:**

This study proposes a contextually relevant model for transitions out of pediatric HIV care in South Africa. Feasible, scalable “protocols” may mitigate risk of worsening posttransition HIV outcomes.

## Abstract 2

Supplemental Digital Content is Available in the Text.

## INTRODUCTION

Adolescents living with HIV demonstrate the poorest health outcomes in care, compared with all other age groups.^1,2^ In resource-limited settings such as sub-Saharan Africa, where the majority reside, adolescents remain largely underserved, and their specific needs in HIV care remain poorly understood—including how those needs change as they become older.^3–5^ As the growing cohort of adolescents on antiretroviral therapy (ART) ages into adulthood, facilitating smooth transition out of pediatric HIV care is essential to ensuring positive treatment outcomes, long-term well-being, and wider public health concerns.^6–9^ Sustained retention in care and ART adherence into early adulthood is crucial for reducing risk of onward transmission, particularly as adolescents become sexually active and enter child-bearing age.^10^

Most published findings on adolescent transition are from high-income countries in North America and Europe, where the dominant transition pathway is from specialized pediatric to specialized adult care.^11–17^ Applicability of this model of care to public health care systems in sub-Saharan Africa is not well documented, but the few studies from the region suggest far greater fluidity in transition process pathways and wide variation in implementation standards.^3,5,18–20^ Without a clear understanding of actual transition experiences in sub-Saharan Africa, application of guidelines from high-income countries may overlook the reality and needs of many adolescents in this setting.

Most transition support guidelines are highly individualized and resourced, including transition readiness assessments or dedicated transition case managers.^11,21^ However, these guidelines may not be relevant or feasible in resource-limited health care systems with high HIV burdens, where greatest efficiency at scale and limited operational demand are necessary. In countries like South Africa, these priorities have necessitated strategies for rapidly scaling up sustainable ART delivery, such as the decentralization of HIV care to primary health care (PHC) clinics.^22^ Since 2010, South Africa has been at the forefront of rapidly decentralizing HIV care, including for children and adolescents living with HIV, which has increased not only care availability but also care-seeking mobility across facilities throughout childhood and adolescence.^19,23,24^ Although South African national guidelines on ART management include protocols for shifting from pediatric to adult ART regimens, there are no formalized guidelines for transitioning adolescents' ART care to the adult setting.^25,26^

In practice, how do such transfers between health care facilities intersect with transition out of specialized pediatric health services? This understanding is critical to ensuring the provision of scalable, sustainable tools and resources that promote successful transition for adolescents living with HIV in high-burden countries.

In this service delivery landscape, we investigate pathways of transition and their associations with HIV outcomes in a longitudinal cohort of adolescents living with HIV in South Africa, using both clinic-based and community-tracing methods to capture the true complexity of pathways in HIV care.

## METHODS

### Participants

The study used a longitudinal, prospective, cohort design with adolescents living with HIV in rural, periurban, and urban locations of a district in the Eastern Cape province, South Africa. The health care system of this region is characterized by high HIV and tuberculosis burdens, limited infrastructure, and significant human resource challenges.^27^ Recruitment took place from March 2014 to September 2015.^28^ Using a list of facilities from the National Department of Health, all health care facilities that provided ART to 5 or more adolescents were identified and included in the study [n = 52, comprising 8 hospital wards, 5 community health centers (CHCs), and 39 PHC clinics]. Each facility's patient register and clinic files were reviewed to identify all adolescents aged 10–19 years who had ever initiated ART, regardless of whether the adolescent was currently engaged in care or had been lost to follow-up (LTFU). Identified adolescents were then traced to more than 180 communities and interviewed at a location of their choice. Overall, 90.1% of eligible adolescents were included in the study. Among those not included, 4.1% of adolescents or their caregivers refused participation, 3.7% were not traceable, 1.2% relocated outside the study area, and 0.9% were unable to participate because of severe cognitive delays.^28^ At 18 months after baseline, adolescents who provided consent (or assent, when <18 year old) were reinterviewed in a second study wave, with 94% retention.

### Data Collection

#### Clinical Patient File Review

At all health care facilities, paper-based and electronic patient files were searched for every study participant. When participants' files were found, data were extracted using a standardized form adapted to the clinical record system in each facility. Data were extracted in 2 rounds, the first covering records from 2014 to 2015 and the second 2016–2017 and included plasma viral load (VL), CD4 cell count, and the World Health Organization staging.

#### Adolescent Interviews

Participants completed tablet-based surveys in 2 study waves with the support of research assistants trained in working with South African adolescents. Surveys included questions about adolescents' lifestyles, health, and health-seeking behaviors and experiences at home, in the community, and in health care facilities. The surveys were developed to be nonstigmatizing and easily understandable through extensive consultation with stakeholders.^28,29^ Surveys were available in both English and Xhosa. These surveys are available at www.mzantsiwakho.co.za.

#### Health care Provider Interviews

Semistructured interviews were conducted with relevant health care staff at all health care facilities. Interviews characterized facilities' service availability and accessibility, human resource capacity, and operational processes.

### Measures

In these analyses, adolescents' patient files were used to identify both clinical care outcomes and mobility across facilities, including transitions out of pediatric care. Adolescent interview data were only used for participants' sociodemographic information.

*Pediatric care* was designated by a dedicated space, day, or time at a facility wherein only children and adolescents received HIV health care services. *Nonpediatric care* was defined as a facility providing generalized care for all ages or one with a dedicated space, day, or time wherein only adults were seen. *Transition out of pediatric care* was identified by having a patient file opened in nonpediatric care, across all facilities, after ART initiation in pediatric care. Among those who transitioned, date of first transition was the date a patient file was first opened in nonpediatric care.

HIV outcomes included viral failure at the most recent VL (HIV-1 RNA ≥1000 copies/mL at most recent VL measurement available across all files),^30^ mortality, LTFU, and posttransition VL change. *Mortality* was ascertained from both clinical records and during community tracing for participant interviews through May 2018. *LTFU* was defined as LTFU recorded in participants' patient files, adjusted for silent transfers found in data collection. Most included health care facilities classified patients as LTFU if they had missed appointments for the past 3 months and were untraceable, when patient tracing was performed by the facility. *“Silent” transfers* were identified when a patient reentered care in a new facility without an official transfer or notification to the former facility of care, where the patient had been deemed LTFU.

*Posttransition VL change* was calculated as the difference between log-transformed posttransition VL and log-transformed pretransition VL. *Pretransition VL* was the last VL available before the first transition out of pediatric care. *Posttransition VL* was the first VL available after the first transition out of pediatric care. Posttransition VL change was dichotomized as log VL change ≤0 and >0, with successful transition considered to be stable or reduced VL posttransition.^31^

### Statistical Analysis

Participants were eligible for inclusion in analyses if they had available patient files. For adolescents without patient files, data on clinical experiences and outcomes were unavailable, and these adolescents were therefore excluded from analyses. Study participants without patient files had been listed on an ART register in at least one health care facility, but actual patient files were missing or unavailable (across paper and electronic forms). An overview of quantitative analyses and their corresponding study sample populations is provided in Table, Supplemental Digital Content 1, http://links.lww.com/QAI/B353.

First, we compared included and excluded adolescents on sociodemographic characteristics and outcomes (age, sex, urban/rural location, mode of infection, and mortality) using χ^2^ and Wilcoxon rank-sum tests for categorical and continuous variables, respectively. Second, using patient file, we characterized adolescent pathways in HIV care by tracing movements across facilities and care types (pediatric/nonpediatric care) over time. Pathways began with the facility care type (pediatric/nonpediatric) and level (hospital/CHC/clinic) at ART initiation. Adolescents who had initiated ART in pediatric care but subsequently received HIV care in nonpediatric settings were considered to have experienced transition out of pediatric care. Third, for the identified transition pathways out of pediatric care, we compared sociodemographic, treatment-related, and outcome variables and tested for groupwise differences using Fisher exact, χ^2^, Kruskal–Wallis, and Wilcoxon rank-sum tests as appropriate.

Fourth, in a cross-sectional analysis, we tested associations between transitioning out of pediatric care and HIV outcomes among included participants using a sequential multivariable regression approach recommended by Hosmer et al.^32^ Variables were removed sequentially: only those significant at *P* < 0.1 were retained in stage 2, with variables significant at *P* < 0.05 retained in stage 3. Final models for each outcome were corrected for false discovery rate using the Benjamini-Hochberg^33^ stepup procedure. Analyses controlled for 10 covariates: (1) age at most recent VL (younger adolescents aged 10–14 years versus older adolescents aged 15–19); (2) sex; (3) residential location at baseline (urban versus rural); (4) horizontal versus vertical mode of infection^34^; (5) year of ART initiation (2000–2009, 2010–2013, and 2013–2017); (6) time on ART (≥2 versus <2 years, measured from ART initiation to date of VL outcome); (7) baseline (first recorded) VL of ≥1000 copies per milliliter; (8) immunologic instability (ever had recorded CD4 cell count of ≤250 cells/mm^3^)^35^; (9) “origin” health care facility level (hospital, CHC, or clinic, defined as most recent facility level before transition out of pediatric care when applicable and ART initiation facility level when no transition was experienced), and (10) any experience of down referral (patient file opened at a lower-level facility after having received care at a higher-level facility). Mortality and LTFU were analyzed for all participants with available patient files, and viral failure at most recent VL only for participants with at least one recorded VL.

Fifth, we tested associations between posttransition VL change and sociodemographic and treatment-related covariates among adolescents who had transitioned, using the sequential multivariable logistic regression approach. Age at first transition out of pediatric care (dichotomized as younger versus older adolescents) was also included as a covariate in this analysis. Only adolescents who had at least 1 VL before and 1 VL after first transition out of pediatric care were included in analyses of posttransition VL change.

Risk of collinearity in regression analyses was assessed using correlation matrices with final models indicating no risk. Finally, thematic analyses of health care provider interviews identified transition support used at included facilities. All statistical analyses were performed using SPSS version 23 (IBM Corp, Armonk, NY).

### Ethical Procedures

Ethical approval was provided by the University of Oxford (SSD/CUREC2/12–21) and the University of Cape Town (CSSR 2013/4; 2017/3), the Eastern Cape Departments of Health and Basic Education, district health management, and management of participating health care facilities. All adolescents of ≥18 years provided voluntary, informed, and written consent for participation in the study, including both participant interviews and access to adolescents' full clinical records across all health care facilities included in the study. All included adolescents of <18 years provided assent, and their primary caregivers provided consent for participation. In cases of low literacy, all information and consent procedures were read aloud in the participant's preferred language. No incentives were provided, but all participants received a certificate of participation, snacks, and a small gift pack including basic items like pencils. Adolescents who chose not to participate were still given snacks and certificates. To minimize the risk of involuntary disclosure and/or stigma, all publicly available study materials were focused on the general health of adolescents in South Africa. To improve reporting and minimize desirability bias, health care provider interviews were conducted by a nurse with more than 2 decades of HIV care experience in the study setting.

## RESULTS

### Cohort Characteristics

Of the 1080 adolescents living with HIV recruited into the study at baseline, patient files were found for 951 (89.8%) as of February 2018. There were no significant differences between included and excluded participants by sociodemographic characteristics or mortality (Table 1). Of the 951 included participants, 54.3% were female, 26.1% were horizontally infected, and the median age at study enrollment was 13 years [interquartile range (IQR), 11–16 years]. Median follow-up time since ART initiation was 7.2 years (IQR, 4.7–9.8 years). At least one VL measurement was available for 92.3% of participants (n = 878).

**TABLE 1.**
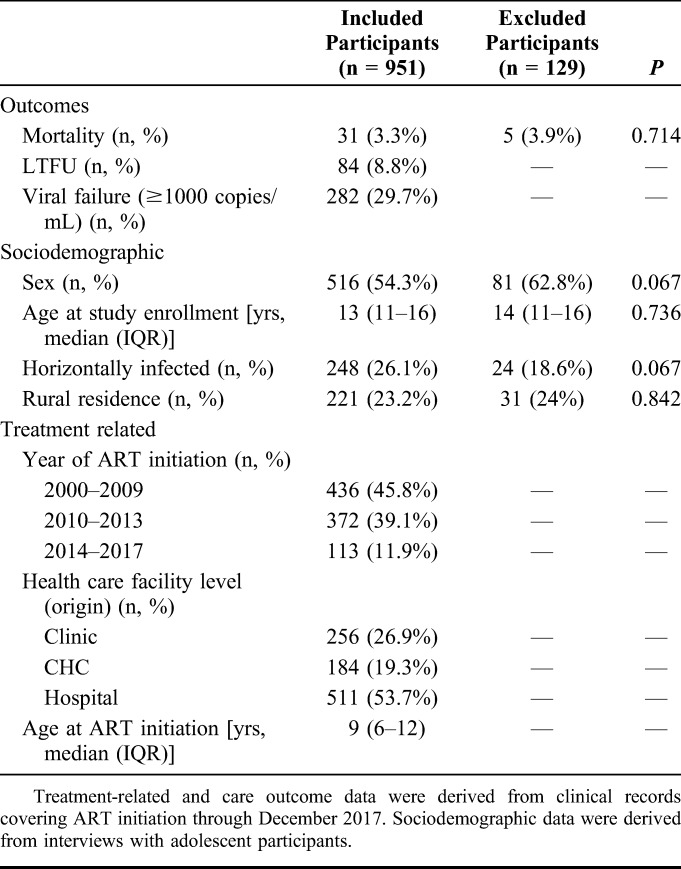
Baseline Demographic, Clinical Characteristics, and Care Outcomes of Included and Excluded Participants

**FIGURE 1.**
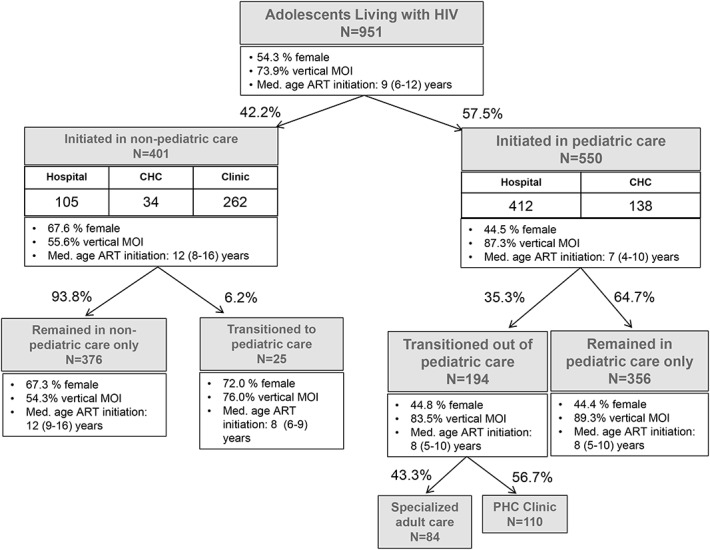
Overview of adolescent pathways in HIV care, by type of care (pediatric/nonpediatric), and health facility (hospital/CHC/PHC clinic). Descriptive characteristics by sex, mode of infection, and median age at ART initiation are provided for each pathway. MOI, mode of infection.

Among included participants, the median age at ART initiation was 9 years (IQR, 6–12 years), with 57.8% (n = 550) having initiated ART in pediatric care at hospitals and CHCs (Fig. 1). Conversely, 42.2% (n = 401) initiated ART in nonpediatric care; a further 93.8% (n = 376) of whom remained exclusively in nonpediatric care. Among those who had initiated ART in pediatric care, 64.7% (n = 356) remained within pediatric hospitals or CHCs and did not transition out of pediatric care. Only 35.3% (n = 194) of adolescents initiated in pediatric care eventually transitioned out of pediatric care, representing 20.4% of the total cohort. Urban living, being on ART for ≥2 years, and a history of immunologic instability were associated with transition (see Table, Supplemental Digital Content 2, http://links.lww.com/QAI/B353).

Of those who had transitioned, 44.8% were female, 16.5% were horizontally infected, and 16.5% resided in rural locations (Table 2). Median age at first transition out of pediatric care was 14 years (IQR, 11–15 years), and median time on ART before first transition was 5.4 years (IQR, 3.3–8.4 years).

**TABLE 2.**
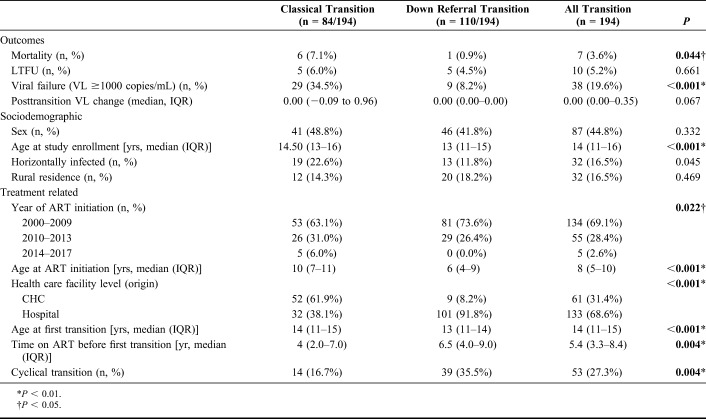
Characteristics of Adolescent Participants by Transition Pathway Out of Pediatric Care

### Transition Pathways

We identified 2 main typologies of transition out of pediatric HIV care: *classical transition to adult HIV care* and *down referral transition to PHC* (Fig. 1).

In the *classical transition* pathway, adolescents transitioned out of pediatric care into specialized adult HIV care in a hospital or CHC, remaining within secondary/tertiary care. In the *down referral transition* pathway, adolescents experienced transition out of pediatric care simultaneously with—and as a result of—down referral to a PHC clinic where they received generalized, nonpediatric HIV care.^24^ Among adolescents who transitioned out of pediatric care, 43.3% (n = 84) experienced *classical transition* and 56.7% (n = 110) *down referral transition*.

Compared with *classical transition*, adolescents who experienced *down referral transition* were younger at study enrollment, ART initiation, and first transition out of pediatric care (Table 2). Adolescents with *down referral transition* were also on ART for longer before first transition and more likely to have transitioned out of pediatric hospital than CHC. Adolescents who experienced *down referral transition* also demonstrated lower rates of viral failure.

Of adolescents who transitioned through either pathway, 27.3% (n = 53) experienced *cyclical transition*. *Cyclical transition* was defined as at least 1 repeated movement between pediatric and nonpediatric care, in which transition out of pediatric care was not a once-off initiating or concluding event. Adolescents who experienced *down referral transition* demonstrated higher rates of *cyclical transition*.

### Associations Between Transition out of Pediatric Care and HIV Outcomes

Overall mortality and LTFU rates in the sample were 3.3% and 8.8%, respectively, although 29.7% of participants demonstrated viral failure at most recent VL. Transitioning out of pediatric care through either pathway was not significantly associated with mortality or LTFU (see Table, Supplemental Digital Content 3, http://links.lww.com/QAI/B353). Transition out of pediatric care was also not associated with the availability of VL data within patient files (see Table, Supplemental Digital Content 4, http://links.lww.com/QAI/B353). VL data were unavailable for only 2.4% (n = 2) of adolescents who experienced *classical transition* and for no adolescents with *down referral transition*.

However, in the final multivariable model, adolescents who experienced *down referral transition* were less likely to demonstrate viral failure at most recent VL [adjusted odds ratio (AOR), 0.25, 95% confidence interval (CI): 0.12 to 0.53; *P* < 0.001], as well as those who transitioned out of a pediatric hospital (AOR, 0.49; 95% CI: 0.33 to 0.73; *P* = 0.001) (Table 3). Older age (AOR, 1.76; 95% CI: 1.25 to 2.48; *P* = 0.001), baseline VL of ≥1000 copies per milliliter (AOR, 3.64; 95% CI: 2.55 to 5.20; *P* < 0.001), and immunologic instability (AOR, 1.64; 95% CI: 1.16 to 2.33; *P* = 0.005) were significantly associated with viral failure at the most recent VL.

**TABLE 3.**
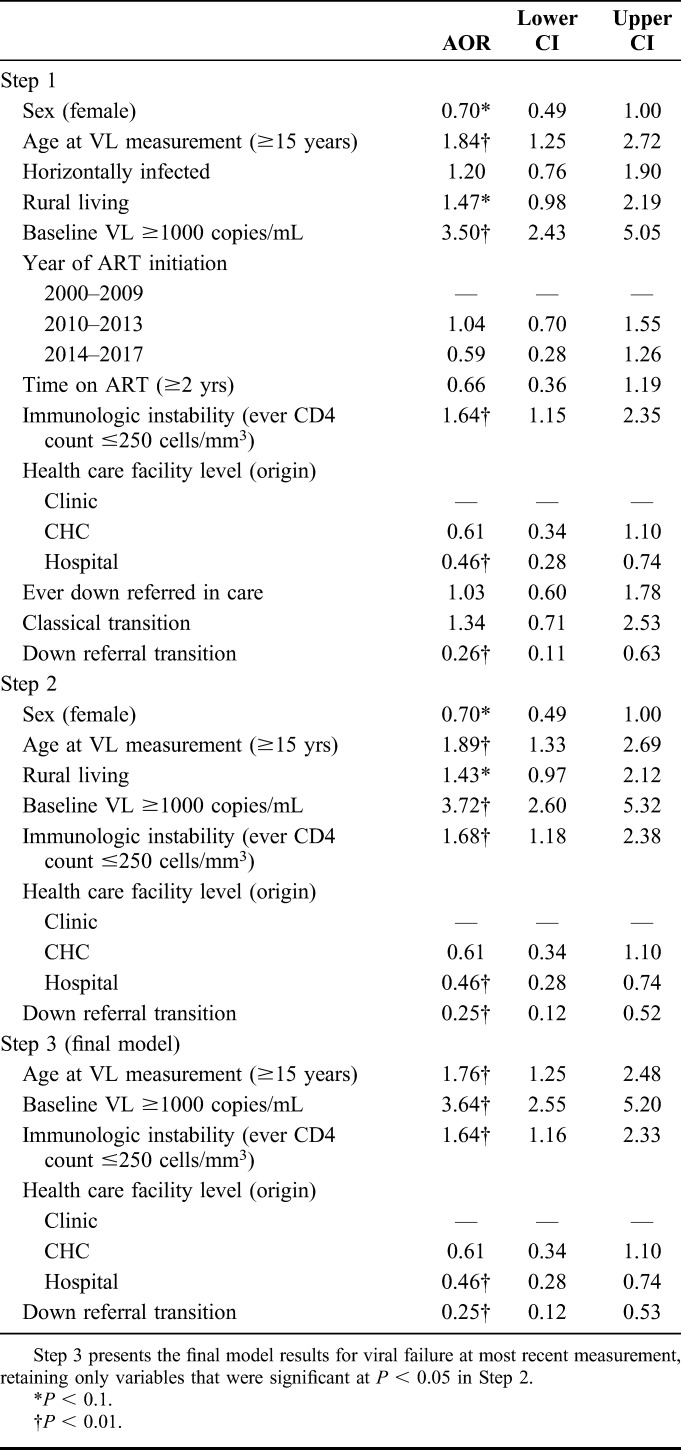
Sequential Multivariable Logistic Regression Analysis Testing Association Between Transition Out of Pediatric Care and Viral Failure

Of the adolescents who transitioned out of pediatric care, 73.7% (n = 143) had a VL available both before and after the first transition event, with a median log VL change of 0.00 (IQR, 0.00–0.35), indicating no significant change in VL posttransition. Median time from first transition to posttransition VL was 2 months (IQR, 0–9 months). There were no significant differences in posttransition VL change between *classical* and *down referral transition* pathways (see Table, Supplemental Digital Content 5, http://links.lww.com/QAI/B353). None of the remaining variables were significantly associated with posttransition VL change.

### Health care Provider Perspectives and Practices of Adolescent Transition

In semistructured interviews at the 13 hospitals and CHCs, health care providers were asked to identify criteria used to determine which adolescents to down refer to PHC clinics. Of these facilities, 10 specified decision-making criteria based on patient health, using 5 major criteria: (1) viral suppression, (2) clinical “stability,” (3) good ART adherence, (4) no treatment complications, and (5) patient willingness to down refer. Viral suppression was the most commonly applied criterion, reported by 8 of the 10 facilities. Clinical “stability” was reported by 5 facilities and described as subjectively determined by the attending health care provider. The remaining 3 criteria were reported by 3 facilities each.

Health care providers at pediatric hospitals described several approaches to mitigate the risk of disengagement from care at the PHC clinic posttransition. These included the provision of continuous counseling and education before transition, with a clear explanation for down referral; offering adolescents a range of clinics closer to home from which they could select their preferred down referral site; contacting PHC clinics for them to anticipate new patients; and obtaining additional contact details from adolescents to check on progress in care after transition.

## DISCUSSION

Using data from routine patient files across 52 public health care facilities in a South African health district, this study characterized the complex reality of adolescent pathways in HIV care across facility care types and levels. Previous studies in sub-Saharan Africa have evaluated adolescent transfers from private care into the public sector or outcomes within specific youth-friendly models of care after transition.^5,21,36^ However, this study is one of the first to quantitatively characterize and evaluate adolescent transitions out of pediatric HIV care within public health care in sub-Saharan Africa.^5,21^

This study suggests that a shift in our current conceptualization of adolescent HIV transition is urgently needed to reflect realities in the sub-Saharan African context, beyond the model of a linear, once-off movement from specialized pediatric care to adult care observed in Europe and North America.^13,14,37^ Using patient files, this study identified 2 distinct typologies of adolescents' transitions out of pediatric HIV care: *classical transition* to adult care and *down referral transition* to PHC. Within a decentralized HIV care model—demanded by high-burden, limited-resource contexts—the majority of adolescents first experienced transition out of pediatric care through *down referral transition*. However, to our knowledge, no previous studies have documented this care trajectory as a transition pathway out of pediatric care. One study in the Western Cape, South Africa, also noted this pattern of care, although it was evaluated in combination with other care transfers, beyond transition out of pediatric care.^19^

As sub-Saharan African countries continue scaling up HIV care, adolescent health care experiences must be considered in the context of structural changes, including decentralized and differentiated care.^38^ For instance, only one-fifth of adolescents in this study had transitioned out of pediatric HIV care, with roughly 40% of adolescents never receiving specialized pediatric care. With South Africa's rollout of nurse-initiated and managed ART in PHC for patients of all ages, a growing number of children and adolescents have been initiated on ART in nonpediatric PHC clinics, even at young ages.^39^ By initiating and continuing to receive care within nonpediatric PHC clinics, these adolescents may never enter pediatric HIV care to transition out of it. As decentralized care is rolled out in similar settings across the region, these findings have important implications for adolescent HIV care provision.

Conversely, 37% of adolescents remained exclusively within pediatric care, which may reflect 3 scenarios. First, younger adolescent participants may not have aged into eligibility for adult HIV care by the end of data collection. Second, through their informal “protocols,” health care providers at included facilities may have delayed transition for specific patients deemed physically or psychosocially unprepared for adult HIV care. This scenario could include patients who were too clinically unstable to down refer out of specialized pediatric care to generalized primary care in clinics. Third, older adolescents may have been instructed to transition out of pediatric care but refused to transfer facilities or care providers.

Applying this expanded and contextually relevant transition model, this study revealed that first transition begins at an earlier age than the reported standard practice of 15 years and older.^5,21^ This finding suggests that programs for facilitating transition must also include younger adolescents. Within South Africa's decentralized care model, there is no age cutoff for down referral eligibility to PHC clinics.^24,38^ Hence, through *down referral transitions*, many adolescents experience transitions out of pediatric care at young ages. In the *classical* model, transitions may be occurring at earlier ages because of patient-initiated transfers to adult care, reflective of patient maturity or preference for care separate from younger pediatric patients.^40^

Furthermore, this study highlights that transition is often not experienced as a once-off event, but rather a *cyclical* movement between pediatric and nonpediatric care after the first transition. In the *classical* model, this cyclical movement may suggest an adolescent patient acclimatizing to care in the nonpediatric setting over time through repeated visits. In the *down referral* model, the cyclical movement may represent up and down referrals as required by fluctuations in the patient's stability or treatment outcomes, such as changing treatment regimens. Interventions and evaluations of transition must account for these potentially diverse treatment trajectories, combining different types and sequences of transitions in HIV care. Such evaluations should consider not only the immediate effects of transitions out of pediatric care but also long-term effects that may emerge years later in late adolescence, when adolescents are particularly at risk for poor HIV care outcomes.^41^

In comparison to all other care trajectories, including remaining within pediatric care, experiencing *down referral transition* was associated with lower probability of viral failure. *Classical transition* was not significantly associated with any HIV outcomes. These findings reflect expected treatment outcomes as a result of decentralization, as only stable patients should be down referred to PHC clinics.^22^ However, adolescents who experienced *down referral transition* remained less likely to demonstrate viral failure, even after adjusting for any experience of down referral (without transition), baseline VL, and immunologic instability. Moreover, median posttransition VL change was not clinically significant. These findings suggest that in the overall adolescent population, regardless of ART initiation site, transition itself did not put adolescents at risk for worse or worsening HIV outcomes.

Our results may be the result of the informal “protocols” for determining which adolescents to transition out of pediatric care, reported by health care providers in the included facilities. Health care providers indicated that adolescents were only transitioned out of pediatric care when the providers were confident in patient stability. Additionally, health care providers described measures taken to facilitate transition, including quality counseling and education for patients and communication with destination facilities. Hence, this study effectively tests the reality of how health care providers have interpreted and implemented clinical guidelines on transition in South Africa's public health sector. Health care providers have been mitigating the risk of negative health outcomes using basic triaging guidelines and transition planning with both patients and destination facilities, which are easily scalable and adoptable in other resource-limited settings.

Study limitations include incomplete patient file and VL availability, with 7.7% of included adolescents lacking any recorded VLs. This study was not able to distinguish specific reasons for missing or infrequent VL recording, which may have resulted from either provider- or patient-side challenges. On the provider end, health care staff may have missed scheduled appointments for blood tests because of inconsistent record-keeping systems; tests may have been ordered but carried out incorrectly, providing invalid results; or test results may have been lost or misfiled.^42,43^ On the patient end, adolescents could be truly LTFU or disengaged from care; regularly picking up medication but unable to wait in long queues for blood tests; or unwilling to take blood tests.

Consequently, VL outcomes could not be estimated for the total sample, and the ability to evaluate long-term outcomes of transition in care was limited. Also, despite intensive data collection within the study area, transfers to health care facilities beyond the health district were not captured. Exclusion of participants without patient files from analyses may have introduced potential bias in reported HIV outcomes. However, with the exception of transfers outside the health district, adolescents without patient files are likely LTFU, and their outcomes would not reflect outcomes in care. Excluding patients without VL data from analyses of the association of transition with viral failure and posttransition VL change may also have introduced bias, but the availability of VL data was not associated with transition out of pediatric care.

However, this study has several key methodological strengths, given the lack of unique national or provincial patient identifiers in the study area. Although most studies of adolescents living with HIV in South Africa have focused on a small number of facilities,^44^ this study looked for clinical records for all participants in all participating facilities. This intensive data collection approach enables the evaluation of adolescents' health as recorded across multiple health care facilities, including unrecorded patient-initiated “silent” transfers to new facilities.^19^

Further analysis is needed to investigate predictors of care outcomes, such as service availability and quality in destination facilities. Additionally, longitudinal analyses of adolescent transitions in care—with greater VL coverage—could better characterize the long-term effects of transition, including for those who transitioned as young adolescents. Finally, future analyses should further investigate and compare the health outcomes of adolescents in nontransitioning pathways, such as adolescents who were kept exclusively within pediatric care or who never received pediatric care. In particular, further work is required to understand why older adolescents may not be transitioning out of pediatric care and how to provide support for these adolescents as they enter young adulthood.

## CONCLUSIONS

As the HIV/AIDS epidemic and its management has evolved in sub-Saharan Africa, research must consider increasingly complex initiation and transition experiences of adolescents, beyond models of care evaluated in high-income countries. This study is unique in documenting—with high rigor—the realities of HIV care trajectories of a large cohort of adolescents living with HIV in a resource-limited setting. With the continued decentralization of HIV care to PHC clinics in sub-Saharan Africa, the findings of this study will bear increasing relevance for the experiences of adolescents in other countries in the region.

## Supplementary Material

SUPPLEMENTARY MATERIAL
