# Supplementary material for: Transition Pathways Out of Pediatric Care and Associated HIV Outcomes for Adolescents Living With HIV in South Africa
Source: J Acquir Immune Defic Syndr. 2019 Jun 20;82(2):166–74. doi: 10.1097/QAI.0000000000002125 (PMC6749967; doi:10.1097/QAI.0000000000002125)
Supplement: SUPPLEMENTARY MATERIAL [file qai-82-166-s001.docx]

**Table, Supplemental Digital Content 1: Overview of study analyses and corresponding relevant sample populations and sizes**

| **Figure/Table** | **Analysis** | **Sample Description** | **Sample size** |
| --- | --- | --- | --- |
| Table 1 | Differences between included and excluded participants, based on patient file availability | All adolescent study participants | n=1080 |
| Figure 1 | Identification of pathways in HIV care, across facility care types and levels | Adolescent study participants with available patient files | n=951 |
| Table, Supplemental Digital Content 2 | Associations with transition out of pediatric care | Adolescent study participants with available patient files | n=951 |
| Table 2 | Comparison between adolescents who experienced down-referral vs. classical transition out of pediatric care | Adolescent study participants who transitioned out of pediatric care | n=194 |
| Table, Supplemental Digital Content 3 | Association between transition and mortality | Adolescent study participants with available patient files | n=951 |
| Table, Supplemental Digital Content 3 | Association between transition and LTFU | Adolescent study participants with available patient files | n=951 |
| Table, Supplemental Digital Content 4 | Association between transition and availability of VL data | Adolescent study participants with available patient files | n=951 |
| Table 3 | Association between transition and viral failure (VL ≥1000 copies/mL) at most recent VL | Adolescent study participants with VL data available in patient files | n=878 |
| Table, Supplemental Digital Content 3 | Association between transition and VL ≥200 copies/mL or VL ≥50 copies/mL at most recent VL | Adolescent study participants with VL data available in patient files | n=878 |
| Table, Supplemental Digital Content 5 | Associations with post-transition VL change | Adolescent study participants who had transitioned out of pediatric care AND who had ≥1 VL available *before* transition and ≥1 VL available *after* transition | n=143 |

LTFU: Lost to follow-up; VL: Viral load

**Table, Supplemental Digital Content 2: Sequential multivariable logistic regression analysis testing associations between baseline sociodemographic and treatment-related variables and transition out of pediatric care.** Step 3 presents the final model results for probability of transition out of pediatric care.

|  | **AOR** | **Lower CI** | **Upper CI** |
| --- | --- | --- | --- |
| **Step 1** | | | |
| Sex (female) | 0.71* | 0.51 | 0.99 |
| Age at study enrollment (≥15 years) | 1.48* | 1.01 | 2.17 |
| Horizontally infected | 0.59* | 0.36 | 0.95 |
| Rural living | 0.55** | 0.36 | 0.84 |
| Baseline VL ≥1000 copies/mL | 1.32 | 0.94 | 1.85 |
| Time on ART (≥2 years) | 5.68** | 2.25 | 14.33 |
| Immunologic instability (ever CD4 count ≤250 cells/mm^3^) | 1.46* | 1.03 | 2.07 |
| **Step 2** | | | |
| Sex (female) | 0.71 | 0.51 | 1.00 |
| Age at study enrollment (≥15 years) | 1.47 | 1.00 | 2.14 |
| Horizontally infected | 0.57* | 0.35 | 0.93 |
| Rural living | 0.54** | 0.35 | 0.83 |
| Time on ART (≥2 years) | 5.67** | 2.25 | 14.30 |
| Immunologic instability (ever CD4 count ≤250 cells/mm^3^) | 1.55* | 1.10 | 2.18 |
| **Step 3 (final model)** | | | |
| Horizontally infected | 0.65 | 0.42 | 1.01 |
| Rural living | 0.54** | 0.35 | 0.82 |
| Time on ART (≥2 years) | 5.53** | 2.20 | 13.92 |
| Immunologic instability (ever CD4 count ≤250 cells/mm^3^) | 1.53* | 1.09 | 2.16 |

AOR: Adjusted odds ratio; ART: Antiretroviral therapy; CI: 95% Confidence interval; VL: Viral load

†*p*<0.1; * *p*<0.05 ; ** *p*<0.01

**Table, Supplemental Digital Content 3: Sequential multivariable logistic regression analysis testing association between transition out of pediatric care and mortality, LTFU, VL** ≥**200 copies/mL at most recent VL, and detectable VL (**≥**50 copies/mL) at most recent VL**

|  | **Mortality (n=31/951)** | | | **LTFU (n=84/951)** | | | **VL** ≥**200 copies/mL (n=283/878)** | | | **VL** ≥**50 copies/mL (n=364/878)** | | |
| --- | --- | --- | --- | --- | --- | --- | --- | --- | --- | --- | --- | --- |
|  | **AOR** | **Lower CI** | **Upper CI** | **AOR** | **Lower CI** | **Upper CI** | **AOR** | **Lower CI** | **Upper CI** | **AOR** | **Lower CI** | **Upper CI** |
| **Step 1** | | | | | | | | | | | | |
| Sex (female) | 0.43† | 0.18 | 1.05 | 0.96 | 0.54 | 1.70 | 0.74† | 0.54 | 1.02 | 0.77† | 0.57 | 1.04 |
| Age at VL measurement (≥15 years) | 3.28* | 1.20 | 8.93 | 0.74 | 0.38 | 1.42 | 1.80** | 1.27 | 2.55 | 1.45* | 1.04 | 2.01 |
| Horizontally infected | 0.22* | 0.05 | 0.91 | 2.60** | 1.32 | 5.09 | 0.93 | 0.61 | 1.43 | 0.99 | 0.66 | 1.48 |
| Rural living | 0.75 | 0.26 | 2.23 | 0.59 | 0.28 | 1.20 | 1.50* | 1.04 | 2.17 | 1.27 | 0.89 | 1.81 |
| Baseline VL ≥1000 copies/mL | 1.24 | 0.52 | 2.94 | 0.94 | 0.52 | 1.70 | 3.41** | 2.45 | 4.74 | 3.11** | 2.28 | 4.24 |
| Year of ART initiation - 2001-2009 - 2010-2013 - 2014-2017 | - 0.71 0.27 | - 0.27 0.04 | - 1.90 1.90 | - 1.08 0.38 | - 0.56 0.12 | - 2.09 1.22 | - 0.97 0.62 | -  0.68 0.30 | - 1.39 1.25 | - 1.11 0.73 | - 0.80 0.37 | - 1.55 1.42 |
| Time on ART (≥2 years) | 0.11** | 0.03 | 0.37 | 0.69 | 0.29 | 1.60 | 0.62 | 0.35 | 1.10 | 0.60† | 0.34 | 1.03 |
| Immunologic instability (ever CD4 count ≤250 cells/mm^3^) | 5.65** | 2.22 | 14.35 | 1.14 | 0.63 | 2.06 | 1.57** | 1.13 | 2.18 | 1.44* | <0.01 | 1.99 |
| Healthcare facility level (origin)   - Clinic - CHC - Hospital | -  0.64  0.67 | -  0.15  0.20 | -  2.83  2.23 | -  0.81  0.18** | -  0.38  0.08 | -  1.71  0.41 | -  0.70  0.46** | -  0.41  0.29 | -  1.20  0.74 | -  0.75  0.48** | -  0.46  0.31 | -  1.24  0.74 |
| Ever down-referred in care | 0.75 | 0.20 | 2.80 | 1.23 | 0.43 | 3.54 | 1.58 | 0.97 | 2.57 | 1.66* | 1.04 | 2.65 |
| Classical transition | 2.55 | 0.66 | 9.85 | 0.68 | 0.22 | 2.10 | 1.03 | 0.57 | 1.85 | 1.12 | 0.64 | 1.96 |
| Down-referral transition | 0.33 | 0.03 | 3.57 | 0.96 | 0.25 | 3.72 | 0.34** | 0.18 | 0.67 | 0.48* | 0.27 | 0.88 |
| **Step 2** | | | | | | | | | | | | |
| Sex (female) | 0.45† | 0.19 | 1.06 | - | - | - | 0.72* | 0.53 | 1.00 | 0.75† | 0.56 | 1.02 |
| Age at VL measurement (≥15 years) | 3.49** | 1.37 | 8.88 | - | - | - | 1.70** | 1.23 | 2.33 | 1.39* | 1.04 | 1.88 |
| Horizontally infected | 0.20* | 0.05 | 0.76 | 2.08* | 1.20 | 3.63 | - | - | - | - | - | - |
| Rural living | - | - | - | - | - | - | 1.57* | 1.10 | 2.25 | - | - | - |
| Baseline VL ≥1000 copies/mL | - | - | - | - | - | - | 3.43** | 2.49 | 4.74 | 3.21** | 2.36 | 4.35 |
| Year of ART initiation - 2001-2009 - 2010-2013 - 2014-2017 | - | - | - | - | - | - | - | - | - | - | - | - |
| Time on ART (≥2 years) | 0.17** | 0.06 | 0.48 | - | - | - | - | - | - | 0.73 | 0.46 | 1.17 |
| Immunologic instability (ever CD4 count ≤250 cells/mm^3^) | 5.58** | 2.28 | 13.64 | - | - | - | 1.62** | 1.18 | 2.24 | 1.46* | 1.07 | 1.98 |
| Healthcare facility level (origin)   - Clinic - CHC - Hospital | - | - | - | -  1.10  0.27** | -  0.59  0.14 | -  2.05  0.54 | -  0.81  0.57** | -  0.50  0.38 | -  1.31  0.85 | -  0.80  0.48** | -  0.51  0.32 | -  1.25  0.71 |
| Ever down-referred in care | - | - | - | - | - | - | - | - | - | 1.78* | 1.14 | 2.75 |
| Classical transition | - | - | - | - | - | - | - | - | - | - | - | - |
| Down-referral transition | - | - | - | - | - | - | 0.48** | 0.28 | 0.82 | 0.44** | 0.25 | 0.78 |
| **Step 3** | | | | | | | | | | | | |
| Sex (female) | - | - | - | - | - | - | 0.72* | 0.53 | 1.00 | - | - | - |
| Age at VL measurement (≥15 years) | 2.94* | 1.20 | 7.21 | - | - | - | 1.70** | 1.23 | 2.33 | 1.32† | 0.99 | 1.77 |
| Horizontally infected | 0.18* | 0.05 | 0.69 | - | - | - | - | - | - | - | - | - |
| Rural living | - | - | - | - | - | - | 1.57* | 1.10 | 2.25 | - | - | - |
| Baseline VL ≥1000 copies/mL | - | - | - | - | - | - | 3.43** | 2.49 | 4.74 | 3.17** | 2.34 | 4.29 |
| Year of ART initiation - 2001-2009 - 2010-2013 - 2014-2017 | - | - | - | - | - | - | - | - | - | - | - | - |
| Time on ART (≥2 years) | 0.19** | 0.07 | 0.52 | - | - | - | - | - | - | - | - | - |
| Immunologic instability (ever CD4 count ≤250 cells/mm^3^) | 5.30** | 2.17 | 12.91 | - | - | - | 1.62** | 1.18 | 2.24 | 1.45* | 1.06 | 1.99 |
| Healthcare facility level (origin)   - Clinic - CHC - Hospital | - | - | - | - | - | - | -  0.81  0.57** | -  0.50  0.38 | -  1.31  0.85 | -  0.83  0.50** | -  0.54  0.34 | -  1.30  0.73 |
| Ever down-referred in care | - | - | - | - | - | - | - | - | - | 1.73* | 1.12 | 2.68 |
| Classical transition | - | - | - | - | - | - | - | - | - | - | - | - |
| Down-referral transition | - | - | - | - | - | - | 0.48** | 0.28 | 0.82 | 0.45** | 0.25 | 0.79 |

AOR: Adjusted odds ratio; ART: Antiretroviral therapy; CHC: Community healthcare center; CI: 95% Confidence interval; LTFU: Lost to follow-up; VL: Viral load

†*p*<0.1; * *p*<0.05 ; ** *p*<0.01

**Table, Supplemental Digital Content 4: Sequential multivariable logistic regression analysis testing association between transition out of pediatric care and availability of VL data**

|  | ≥**1 VL recorded in patient file**  **(n=878/951)** | | |
| --- | --- | --- | --- |
|  | **AOR** | **Lower CI** | **Upper CI** |
| **Step 1** | | | |
| Sex (female) | 0.34* | 0.13 | 0.90 |
| Age at study enrollment (≥15 years) | 0.49 | 0.17 | 1.40 |
| Horizontally infected | 0.28* | 0.10 | 0.78 |
| Rural living | 2.18† | 0.89 | 5.34 |
| Year of ART initiation - 2001-2009 - 2010-2013 - 2014-2017 | - 0.26 0.06** | - 0.05 0.01 | - 1.33 0.32 |
| Immunologic instability (ever CD4 count ≤250 cells/mm^3^) | 0.32** | 0.15 | 0.66 |
| Healthcare facility level (origin)   - Clinic - CHC - Hospital | -  1.31  1.70 | -  0.50  0.52 | -  3.46  5.50 |
| Ever down-referred in care | 0.93 | 0.21 | 4.12 |
| Transition out of pediatric care (classical or down-referral) | 1.85 | 0.34 | 9.98 |
| **Step 2** | | | |
| Sex (female) | 0.29* | 0.11 | 0.75 |
| Age at study enrollment (≥15 years) | - | - | - |
| Horizontally infected | 0.18** | 0.07 | 0.43 |
| Rural living | 1.94 | 0.80 | 4.71 |
| Year of ART initiation - 2001-2009 - 2010-2013 - 2014-2017 | -  0.22†  0.04** | -  0.05  0.01 | -  1.01  0.16 |
| Immunologic instability (ever CD4 count ≤250 cells/mm^3^) | 0.33** | 0.16 | 0.67 |
| Healthcare facility level (origin)   - Clinic - CHC - Hospital | - | - | - |
| Ever down-referred in care | - | - | - |
| Transition out of pediatric care (classical or down-referral) | - | - | - |
| **Step 3** | | | |
| Sex (female) | 0.30* | 0.12 | 0.77 |
| Age at study enrollment (≥15 years) | - | - | - |
| Horizontally infected | 0.17** | 0.07 | 0.42 |
| Rural living | - | - | - |
| Year of ART initiation - 2001-2009 - 2010-2013 - 2014-2017 | -  0.21  0.04** | -  0.05  0.01 | -  0.99  0.17 |
| Immunologic instability (ever CD4 count ≤250 cells/mm^3^) | 0.34** | 0.17 | 0.68 |
| Healthcare facility level (origin)   - Clinic - CHC - Hospital | - | - | - |
| Ever down-referred in care | - | - | - |
| Transition out of pediatric care (classical or down-referral) | - | - | - |

AOR: Adjusted odds ratio; ART: Antiretroviral therapy; CHC: Community healthcare center; CI: 95% Confidence interval; LTFU: Lost to follow-up; VL: Viral load

†*p*<0.1; * *p*<0.05 ; ** *p*<0.01

**Table, Supplemental Digital Content 5: Sequential multivariable logistic regression analysis testing associations between baseline sociodemographic and treatment-related variables and post-transition VL change (log VL change ≤0)**

|  | **AOR** | **Lower CI** | **Upper CI** | ***P*** |
| --- | --- | --- | --- | --- |
| Sex (female) | 1.81 | 0.81 | 4.04 | 0.148 |
| Age at post-transition VL measurement (≥15 years) | 0.40 | 0.06 | 2.63 | 0.340 |
| Horizontally infected | 2.33 | 0.64 | 9.49 | 0.200 |
| Rural living | 1.21 | 0.42 | 3.5 | 0.730 |
| Year of ART initiation - 2001-2009 - 2010-2013 - 2014-2017 | - 1.79 0.98 | - 0.70 0.10 | - 4.61 9.40 | - 0.225 0.987 |
| Time on ART (≥2 years) | 0.87 | 0.18 | 4.17 | 0.860 |
| Age at first transition (≥15 years) | 0.68 | 0.09 | 5.29 | 0.710 |
| Origin healthcare facility (hospital) | 0.81 | 0.25 | 2.63 | 0.73 |
| Cyclical transition | 0.73 | 0.28 | 1.93 | 0.520 |
| Down-referral transition | 1.50 | 0.56 | 4.05 | 0.420 |

AOR: Adjusted odds ratio; ART: Antiretroviral therapy; CI: 95% Confidence interval; VL: Viral load
